# The safety and efficacy of Glubran 2 as biliostatic agent in liver resection

**DOI:** 10.1186/s13027-021-00358-3

**Published:** 2021-03-16

**Authors:** Renato Patrone, Vincenza Granata, Andrea Belli, Raffaele Palaia, Vittorio Albino, Mauro Piccirillo, Roberta Fusco, Fabiana Tatangelo, Guglielmo Nasti, Antonio Avallone, Francesco Izzo

**Affiliations:** 1 PhD ICTH, University Federico II, Via Sergio Pansinin 5, 80131 Naples, Italy; 2 Department of Support to Cancer Pathways Diagnostics Area, Radiology Unit, “Istituto Nazionale Tumori IRCCS Fondazione Pascale – IRCCS di Napoli”, Naples, Italy; 3 Radiology Division, “Istituto Nazionale Tumori IRCCS Fondazione Pascale – IRCCS di Napoli”, Naples, Italy; 4 Department Corp-S Care and Research of Cancer of the Abdominal District, Hepatobiliary Unit, “Istituto Nazionale Tumori IRCCS Fondazione G. Pascale – Napoli”, Naples, Italy; 5 Department of Support to Cancer Pathways Diagnostics Area, Pathology and Cytopathology Unit, “Istituto Nazionale Tumori IRCCS Fondazione G. Pascale – IRCCS Napoli”, Naples, Italy; 6 Department Corp-S Care and Research of Cancer of the Abdominal District, Oncology Unit, “Istituto Nazionale Tumori IRCCS Fondazione G. Pascale – IRCCS Napoli”, Naples, Italy

**Keywords:** Liver metastases, Bile duct injury, Hepatic resection, Chemotherapy, Efficacy

## Abstract

**Background:**

Biloma, an encapsulated collection of bile outside the biliary tree, supported by a predominantly iatrogenic biliary fistula, and bile likeage are two of the most important surgical complications after liver resection. We, hypothesized to conduct a project aimed to prevent, or reduce, the formation of biloma or biliary fistula applying on the hepatic resection area the cyanoacrylate glue (Glubran2).

**Methods:**

We searched in our surgical database all patients underwent liver resection for mCRC from January 2013 to December 2018 and we found a total of 510 patients. 205 patients for Group A (study population: included patients in which we have used Glubran2 during surgical procedure) and 113 patients for Group B (control group), were enrolled.

**Results:**

In both Groups no patients died during hospitalization and the 30-day mortality was 0 %. During follow-up in Group A, a biliary fistula was found in 2 patients (1 %) versus 3 patients in the Group B (2,6 %). In patients enrolled in Group A no adverse event were reported relate to the use of Glubran2.

**Conclusions:**

It is possible to affirm that the use of Glubran2 as biliostatic agent after liver resection is useful to prevent bile leakage complication and biloma formation and its use demonstrated to be safe and feasible during liver surgery.

## Background

The only curative option for patients with colorectal liver metastases (mCRC) enabling 5-year overall survival rates of 50 %, is hepatic resection [1, 2]. Effective oxaliplatin- and irinotecan-based chemotherapy protocols associated with targeted agents have significantly improved response rates, conversion to resectability and long-term survival in mCRC patients [1]. However, nevertheless the benefit of neoadjuvant chemotherapy, there are a number of chemotherapy-effects that have an influence on surgical morbidity. The chemotherapy-related complications, steatosis, chemotherapy-associated steatohepatitis (CASH) and sinusoidal obstruction syndrome (SOS), might impair the hepatic parenchyma, thus reducing the functionality and influencing the outcome following resection [1–3]. One of the most serious complication reported in Literature, related to liver surgery, are the biliary system iatrogenic injury with an important rate from 3.6 to 17 % depending on several clinical risk factor [3–7]. Due to an increase of postoperative mortality rates related to biliary complications the use of topical hemostatic agents have been recommended [4, 5, 8–10]. Nonetheless, in Literature, only few study focused attention on the use of these agents to prevent complication related to liver resection [11–15]. In this study, the topical hemostatic agent used was a new synthetic cyanoacrylate glue called Glubran2, tested in various surgery with promising results [16–18]. Our primary endpoint is to test the safety and feasibility of Glubran2 during surgical liver resection in patients with mCRC previously treated with chemotherapy; as secondary endpoint we selected the utility of this agent to prevent biloma or biliary fistula to assess its biliostatic effect.

## Methods

### Study population

We searched, in the surgical database of the National Cancer Institute of Naples, all patients who underwent liver resection for mCRC from January 2013 to December 2018 and we found a total of 510 patients. From this total number we divided patients in two groups: Group A (study population) included patients in which we have used Glubran2 after liver resection, and Group B (control group).

 This retrospective study was approved by the Ethical Committee of the National Cancer Institute “G. Pascale Foundation - IRCCS” of Naples and the requirement for patient informed consent was waived.

The inclusion criteria for the study population and control group were: *(a)* patients who had pathologically proven mCRC; *(b)* patients who had undergone imaging studies within 1 month to surgical procedure; and *(c)* patients who had been subjected to the same neo-adjuvant treatments. The exclusion criteria were: *(a)* discrepancy between the pre-surgical diagnosis and the pathologically confirmed diagnosis, *(b)* no available follow-up imaging studies.

 During the study period, 205 patients for Group A and 113 patients for Group B, were enrolled in the study that fulfilled the inclusion criteria.

Characteristics of patients from both groups are summarized in Table 1.

**Table 1:** Datation regarding the MR imaging pps

	mCRC patients (no.=205)	Control patients (no.=113)	***P*** value
***Demographics***			
Gender	Men 89 (43.4%)	Men 64 (56.6%)	0.89
	Women 116 (56.6%)	Women 49 (43.4%)	0.89
Age	Mean, 56 years	Mean, 48 years	0.74
	Range, 33-80 years	Range, 35-78 years	
***Primary cancer site***			
Colon	94 (45.9%)	52 (46%)	0.92
Rectum	111 (54.1%)	61 (54%)	0.92
***History of chemotherapy***	205 (100 %)	113 (100%)	0.99
*Chemotherapy protocol*	mFOLFOX6 (5-fluorouracil, leucovorin, oxaliplatin) plus bevacizumab 205 (100%)	mFOLFOX6 (5-fluorouracil, leucovorin, oxaliplatin) plus bevacizumab 113 (100%)	
***Liver metastases***			
Number	1075	452	
	mean 4 per patient	mean 5 per patient	
	range 1-7 per patient	range 2-6 per patient	
Largest diameter	mean 32 mm	mean 28 mm	
	range 8-64 mm	range 10-54 mm	
**Complications**			
Biloma	27 (13%)	18 (16%)	**0.054**
Bile leakage	2 (1%)	3 (2.6%)	**0.001**

### Chemotherapy protocol

Both groups of patients received neoadjuvant mFOLFOX6 (5-fluorouracil, leucovorin, oxaliplatin) plus bevacizumab. mFOLFOX6 was administered IV every 14 days with oxaliplatin 85 mg/m-2 by infusion on day1, followed by leucovorin 200 mg/m-2 infusion on day 1, followed by 5-fluorouracil 400 mg/m-2 bolus on day 1, and 5-fluorouracil 2400 mg/m^− 2^ 46-h continuous infusion. The antiangiogenic drug Bevacizumab was administered every 14 day-sat 5 mg/kg by IV infusion over 90 min at the first cycle, and then, if adequately tolerated, over 60 min. The treatment of mFOLFOX6 plus bevacizumab was administered for a total of 6 cycles.

### Surgical procedure

All resections were initiated with curative intent. Surgical exploration and intra-operative ultrasound were performed in all cases to detect occult tumors and to plan appropriate resections. Resections of all metastatic sites were executed as anatomic or non-anatomic resections with the goal of maximal parenchymal preservation by non-anatomic resection. Dissection was accomplished using SonaStar by Misonix, allowing precise, non-anatomic resections. Major hepatectomy was defined as resection of three or more liver segments. Patients with synchronous colorectal and liver tumor at the time of presentation were assessed for feasibility of single stage combined colon and liver resection by the multidisciplinary team. In general, younger patients in good general condition and no significant comorbidity conditions were deemed candidates for single stage combined liver and colon resections.

### Haemostatic agents

Glubran2 is a synthetic surgical glue, (CE Mark) certificated for internal and external use, with haemostatic, adhesive, sealer, and bacteriostatic properties. When used in moist environment, it quickly polymerizes into a thin elastic film which has high tensile strength and firmly adheres to the anatomy of the tissue on which it is applied. Once it is polymerized, Glubran2 acts as a bio inert material. We used 1 package of 1mL Glubran2 for each patient.

### Lesion confirmation: reference standard

Two pathologists, specialized in the liver, performed histopathologic analysis of resected specimens. Lesion confirmation was based on the pathologic diagnosis of surgically resected liver specimens. The resected specimens were processed and then sectioned with a 5-mm slice thickness. All tumor samples were stained with hematoxylin and eosin coloration. Immunohistochemistry stains were obtained to confirm the intestinal origin of the metastatic lesions. The panel of immunohistochemical markers included cytokeratin 7, cytokeratin 20, and CDX2. The histopathological report included the pushing or infiltrating growth and the presence or absence of tumor budding and/or fibrosis and necrosis.

### Follow‐up

Al patients underwent to US and CT at 1 month post surgical resection. A MDCT study was performed at 3th, 6th and 12th month. MRI study was a problem-solving tool for patients with suspicious of recurrence disease or in which a complication was detected.

OS was defined as the interval (in months) from the date of partial hepatectomy to the date of death.

### MDCT protocol

CT studies were performed with a 64-detector row scanner (Optima 660, GE Healthcare, USA), using the following scanning parameters: 120 kVp, 100–470 mAs (NI 16.36) and 2.5-mm slice thickness. Liver protocol study in our Cancer Center includes a quadruple phases contrast study with an unenhanced, an arterial, a portal/venous, and equilibrium phases. Images acquisition in the arterial phase is started after attenuation in the descending aorta reached 120 HUs, measured with the bolus tracking method. For the portal/venous phase, the images were acquired 33 s after the arterial phase. For the equilibrium phase, images were acquired 180 s after the contrast medium injection.

### MR Imaging Protocol

MR studies were performed by using a 1.5T scanner (Magnetom Symphony, with Total Imaging Matrix Package, Siemens, Erlangen, Germany) with an 8-element body coil and a phased array coil. Detailed information regarding the MR study protocol is summarized in Table 2. A standard dose (0.025 mmol/kg) of gadoxetic acid (Primovist, Bayer Healthcare, Berlin, Germany) was injected at a rate of 1.0 mL/s by using a power injector (Spectris Solaris EP; Medrad, Warrendale, Pa) followed by a 30-mL saline flush. Arterial phase images were acquired 7 s after contrast medium arrival at the thoracic aorta by using an MR fluoroscopic monitoring system. Thereafter, portal venous phase, transitional phase, and hepatobiliary phase (HBP) were obtained 60 s, 3 min, and 20 min after contrast medium injection, respectively.

**Table 2:** Detailed information regarding the MR imaging parameters

Sequence	Orientation	TR/TE/FA (ms/ms/deg.)	AT (min)	Acquisition Matrix	ST/Gap (mm)	FS
Trufisp T2-W	Coronal	4.30/2.15/80	0.46	512 × 512	4 / 0	without
HASTE T2-W	Axial	1500/90/170	0.36	320 × 320	5 / 0	Without and with (SPAIR)
HASTE T2w	Coronal	1500/92/170	0.38	320 × 320	5 / 0	without
In-Out phase T1-W	Axial	160/2.35/70	0.33	256 × 192	5 / 0	without
DWI	Axial	7500/91/90	7	192 × 192	3 / 0	without
Vibe T1-W	Axial	4.80/1.76/12	0.18	320 × 260	3 / 0	with (SPAIR)

Note. *TR *Repetition time, *TE *Echo time, *FA *Flip angle, *AT *Acquisition time, *ST *Slice thickness, *FS*  Fat suppression, *SPAIR *Spectral adiabatic inversion recovery

### CEUS protocol

CEUS was always preceded by a careful US survey, assessing the size and appearance of the lesion/s. This baseline assessment was done to appropriately choose the liver area or areas to be particularly focused in the forthcoming contrast-enhanced part of the US study. In all cases, a separated injection was performed for each liver lobe. For both injections, the arterial phase assessment was focused on any known lesion at baseline US. CEUS was performed as a low- mechanical index, double-split mode, real-time modality. We employed a Technos MyLab 70 XVG and MyLab Twice scanner (Esaote, Genoa, Italy), injecting 2.4 ml of a sulfur hexafluoride-based contrast medium (SonoVue, Bracco, Milan, Italy) per each liver lobe. After the injection, the radiologist focused the sonographic field of view on the parenchymal area of interest, waiting for the microbubble’s arrival. Thereafter, he/she moved the transducer to explore the remaining parenchyma of each lobe, with special reference to the resected area.

### Biloma and bile leakage definition, diagnosis and management

Following scientific Literature we considered biloma as an encapsulated collection of bile outside the biliary tree and within the abdominal cavity and bile leakage as a postoperative loss of fluid bile via abdominal drains after liver surgery [19, 20]. We diagnosed and divided bile leakage in grade A, B and C based on the impact of this complication on patients’ clinical management. [20]

### Surgical complications

Surgical Complications were classified according to Clavien Dindo et al. [21].

### Statistical analyses

Each continuous variable was expressed in terms of median value ± range while each variable categorical was summarized by frequencies and percentages. Chi square test was performed to assess statistically significant difference between percentage values. Mann Whitney non parametric test were used to compare a continuous variable between 2 groups. A *p* value < 0.05 was considered statistically significant.

All statistical analysis was performed with SPSS for Windows (Version 23.0; SPSS Inc, Chicago, Ill).

## Results

We analyzed a total of 318 patients: Group A with 205 patients and 1036 pathologically proven lesions (mean tumor size: 32 mm; range 8–64 mm) and Group B with 113 patients and 452 mCRC pathologically proven lesions (mean tumor size: 36 mm; range 11–59 mm).

In the Group A we performed 60 lobectomy, 43 meso-hepatectomy, 48 bi-segmentectomy (73 % major hepatectomy) and 54 segmentectomy or other liver resection (wedge/metasasectomy) (2 in seg I, 3 in seg II, 4 in seg III, 10 in seg IV, 9 in seg V and 8 in seg VI, 18 in seg. VII). Twenty-five patients underwent single stage combined liver and colon resections.

The average hospitalization time was 8 days (7–16). No major complications occurred during surgical procedures. No patients died during hospitalization and the 30-day mortality was 0 %. During follow-up in 27 patients (13 %) was reported a Biloma and in 2 patients (1 %) a bile leakage grade B was detected. No adverse events were reported regarding the use of Glubran 2.

About Group B (113 patients), 56 patients underwent major hepatectomy (49,5 %), 40 liver segmentectomy (35.5 %), 17 wedge procedure or metasasectomy (5 in seg II, 2 in seg III, 3 in seg IV, 2 in seg V and 5 in seg VI). Seventeen patients underwent single stage combined liver and colon resections.

The average hospitalization time was 10 days (5–14). No major complications occurred during surgical procedures. No patients died during hospitalization and the 30-day mortality was 0 %.

During follow-up 18 patients (16 %) showed presence of biloma and in 3 patients (2,6 %) a bile leakage was detected (2 grade B, 1 grade C).

A new hepatic lesions were identified (mean time 5 months) in 32 patients (15,6 %), 13 in group A and 19 in group B.

## Discussion

The rationale behind our study is the polymerization of cyanoacrylate glue when it is in contact with blood and tissues owing to the presence of ions and proteins. Nowadays the solid polymer created by this reaction, has demonstrated to be safe and useful in several use during surgical clinical practices.

In surgery for ventral hernia repair, Glubran2 permitted a suturless fixation mesh, in bariatric surgery showed to be effective to prevent gastric fistulas or suture line dehiscence (leaks), in endovascular surgery many scientific articles had described it’s use to stop bleeding in elective and in emergency and new applications are investigate in colorectal and thoracic surgery, to prevent anastomotic and air leak [22–28].

Our study is focused on biliostatic effect of cyanoacrylate glue but, in Literature, is possible to find articles that analyzed or compare hemostatic or sealant agent to prevent bile leakage and hemorrhagic events. López-Guerra D et al., in 2019, compare the use of two different fibrin sealant patches during liver surgery. Contrary to ours, this study included benign patients, patients without pre-operative chemotherapy, HCC and mCRC and concluded with no superiority between different fibrin patches in post-operative complications not focusing attentions on bile leakage [18].

Likewise others papers compare different agent both on prevent bleeding and bile leakage and, similarly to López-Guerra D et al., their population study included not only mCRC patients [29–31].

Also Briceño J et al., evaluating a fibrin sealant, concluded advising the use of this agent during liver surgery due to the decrease moderate and severe postoperative complications with no clarification on bile leakage impact [29].

Precisely for this, our study acquire relevance because this is the first study that assesses the safety and efficacy of Glubran2 as a biliostatic agent, at the best of our knowledge, focusing attention to prevent bile post-opertive complications.

This project was inspired by the evolution of neoadjuvant chemotherapy for colorectal liver metastases. Thanks to the use of new drugs, especially anti-angiogenetic monoclonal antibody added to usual chemotherapy, has become possible to treat surgically patients with more curative intent due an increase response rates conversion to resectability and long-term survival. This is a very important result considering that hepatic resection is the only curative option for patients with colorectal liver metastases (mCRC) [1]. However, we need to consider also the neoadjuvant chemotherapy influence on surgical morbidity. The chemotherapy-related complications, steatosis, chemotherapy-associated steatohepatitis (CASH) and sinusoidal obstruction syndrome (SOS), might impair the hepatic parenchyma, thus reducing the functionality and influencing the outcome following resection [1, 2]. Despite the results of same studies demonstrated that neoadjuvant chemotherapy did not impair outcomes of liver resections for mCRC, in our experience we reported an increase of complications rates in this type of patients [32]. The most peculiar and important complication after liver resection is bile leakage with an incidence that is reported between 3.6 and 17 % [33]. In our two groups, we evaluate and match age, sex, site of primary cancer, chemotherapy protocol, duration of chemotherapy and type of liver resection. All patients underwent to US and MDCT at one month post resection and MDCT at 3th, 6th and 12th month for the first post-operative year. In the Group A we performed major hepatectomy in 73 % of patients versus 49.5 % in Group B. Several researches have shown that risk of biliary complications increase with the complexity of surgical procedure [34]. Patients enrolled in Group A were compared with patients in Group B; biloma was reported in 13 % (group A) vs. 16 % (group B) and bile leakage in 1 % (Group A) vs. 2.6 % (group B) (*P*-value < 0,001). All patients underwent liver resection using the same surgical open or laparoscopic approach, uniform technique and energy devices. In literature is possible to find numerous study that compared different types of advanced energetic devices and their haemostatic effect, their lateral spread damage in many tissues and no one demonstrated better result over others [35]. In all our patients, in booth groups, we used only Harmonic Scalpel in order to minimizing the intraoperative bias.

During the follow up, no patients died but a new hepatic lesions were found in 32 patients (15,6 %): 13 in Group A and 19 in Group B.

We observed no adverse events regarding the use of Glubran2. Analyzing our results, we could state that Glubran2 is a safe and efficacy biliostatic agent useful to prevent bile leakage complication after liver resection. Our results are similar to others that have shown that Glubran2 is a safe and effective hemostatic agent [20, 34, 36, 37].

The current study had several limitations: data collected derived from only one cancer centre, and a small semple size enrolled in this study may have influence the conclusion. In addition, this is a retrospective study. Therefore, further perspective multicentre analyses including more patients were needed to validate the prognostic significance of these results.

## Conclusions

Answering to our primary end-point, it is possible to affirm that Glubran2 is a safe and feasible biliostatic agent useful to prevent bile leakage complication and biloma formation after liver resection.

## Data Availability

The datasets used and/or analysed during the current study are available from the corresponding author on reasonable request.

## Abbreviations

mCRC: Colorectal liver metastases
CASH: Chemotherapy-associated steatohepatitis
MDCT: Multiple detector computed tomography
CT: Computed tomography
CEUS: Contrast Enhanced Ultrasound
SOS: Sinusoidal obstruction syndrome
